# Digital Evaluation of Orbital Cyst Associated with Microphthalmos: Characteristics and Their Relationship with Orbital Volume

**DOI:** 10.1371/journal.pone.0157819

**Published:** 2016-06-17

**Authors:** Ying Cui, Yue Zhang, Qinglin Chang, Junfang Xian, Zhijia Hou, Dongmei Li

**Affiliations:** 1 Beijing Tongren Eye Center, Capital Medical University, Beijing, China; 2 Department of Ophthalmology, Beijing Tongren Hospital, Capital Medical University, Beijing, China; 3 Beijing Ophthalmology & Visual Science Key Lab, Capital Medical University, Beijing, China; 4 Department of Medical Imaging Center, Beijing Tongren Hospital, Capital Medical University, Beijing, China; Sun Yat-sen University, CHINA

## Abstract

**Purpose:**

To study the characteristics of orbital cyst associated with microphthalmos in a group of Chinese patients, and to analyze the relationship between orbital cyst and orbital volume.

**Design:**

Cross-sectional comparative study.

**Participants:**

120 patients who were diagnosed as unilateral clinical blind microphthalmos, in which 20 patients had orbital cyst in the affected eye.

**Method:**

Participants had computed tomography (CT) scan. CT images were analyzed with a computer-aided software.

**Main Outcome Measures:**

Volume and position of orbital cyst, microphthalmic to contralateral ratio (MCR) of orbital volume, height and depth and orbital rim displacement.

**Results:**

38.1% of the cysts located anterior to or at the equator of the globe, 75% of which located infratemporally and all of which were outside the muscle cone. Most (84.6%) of the posterior cysts were inside the muscle cone. The anterior cysts were larger than the posterior cysts (p = 0.005). MCR of orbital volume (p<0.001), height (p = 0.004) and width (p = 0.043) were significantly higher in patients with orbital cyst than controls. For patients with orbital cyst, larger cyst-plus-globe volume of the affected eye was associated with higher MCR of orbital volume (r = 0.630, p = 0.003). Patients with large cyst-plus-globe volume had higher MCR of orbital volume (p = 0.002), height (p = 0.014), width (p = 0.005) and depth (p = 0.002) and less displacement in inferior (p = 0.004) orbital rim, compared with patients with small cyst-plus-globe volume, but the differences between patients with small cyst-plus-globe volume and patients without cyst were not significant.

**Conclusions:**

Microphthalmic eyes with large cyst-plus-globe volume showed better similarity with the contralateral eyes, comparing with microphthalmic eyes without orbital cyst or with small cyst-plus-globe volume. It suggested that the existence of orbital cysts (especially large cysts) in microphthalmic eyes might play a positive role in the orbital development.

## Introduction

Microphthalmos with orbital cysts is a rare, severe malformation resulted from defective closure of the embryonic fissure and over-growth of the inner layer of the optic cup. There have been many case reports about this congenital anomaly since its first report in 1858.[1–10] McLean et al[11] studied the management of orbital cysts present in 34 patients with microphthalmos and anophthalmos. Chaudhry et al[12] analyzed the feature and management of 23 patients who were diagnosed as microphthalmos with orbital cyst. Both of them focused mainly on the management of the cysts, while the characteristics of the cysts and, more importantly, their relationship with orbital volume were not fully discussed.

One of the most important esthetic problems of microphthalmic patients is the hypoplasia of the affected orbit and hemifacial deformity. In our clinical practice, we found that some of the microphthalmic patients with orbital cysts showed less retardation of development in the affected orbit, compared with patients without cyst. It occurred to us that the orbital cysts, which serve as volume replacement and simulate the orbital implants to some extent, might also play a positive role in orbital expansion. In our previous study, computerized tomography (CT) and iPlan Cranial software were used to measure orbital parameters and allowed us to evaluate the orbital development in Chinese children with congenital microphthalmos.[13] With CT images and iPlan Cranial software, accurate measurements of the cyst and orbit could be collected, making it possible for quantitative analysis of the relationship between orbital volume and the cyst.

## Materials and Methods

### Participants

This clinical comparative study included 120 patients who were diagnosed as unilateral clinical blind microphthalmos in Beijing Tongren Hospital between July 2009 and January 2015 in a consecutive manner, in which 20 patients were found to have orbital cyst in the affected eye. All the patients had finished CT scanning. The study was approved by the Ethics Committee of Beijing Tongren Hospital and adhered to the provisions of the Declaration of Helsinki for research involving human subjects. Written informed consent was obtained from all adult participants. For the minors/children, written informed consent was obtained from their parents.

Clinical blind microphthalmos was defined as 1) markedly decreased ocular size to the contralateral eye (unilateral cases)/ markedly decreased axial length to normal axis adjusted for age (bilateral cases); 2) lack of normal globe structure on imaging examinations (CT, MRI or ultrasound); 3) no light perception. Exclusion criteria were secondary microphthalmos (persistent hyperplastic vitreous, traumatic history, etc) and diseases that need to be differentiated from microphthalmos with orbital cyst (congenital cystic eye, dermoid cyst, teratoma, encephalocele, meningocele, etc).

Examinations included visual acuity, general slit-lamp examinations, CT scan, and a detailed questionaire. Each of the newly diagnosed microphthalmic patients was consulted by a pediatrician to find any systemic abnormalities. Data were collected before any treatment.

### CT examination and volume measurement

Each patient was scanned with a Brilliance 64-channel multidetector CT scanner (Philips Medical Systems Inc, Cleveland, Ohio, USA) with the orbitomeatal line as baseline. Scanning parameters for adults were 120kV, 250mA, 16×0.625mm detector collimation, with a pitch of 0.563. Images were reconstructed on a 512×512 pixel matrix at a thickness of 0.67 mm. To minimize the radiation exposure in children, we adhered to the ALARA (as low as reasonably achievable) principle[14] by adjusting the parameters such as peak kilovoltage and tube current to lower level (100kV, 93.8mAs), and limiting the scans to the orbital region. The images were imported to the iPlan Cranial software (Version 2.5; BrainLAB, Munich, Germany), which generated 3-dimensional (3D) reconstructions of the orbit and enabled digital measurements of orbital volume, width, height, depth and orbital rim displacement. The volumes, pathlines and orbital rim displacement were measured with techniques described in our previous study.[13]

#### Orbital volume measurement

**Fig 1:**
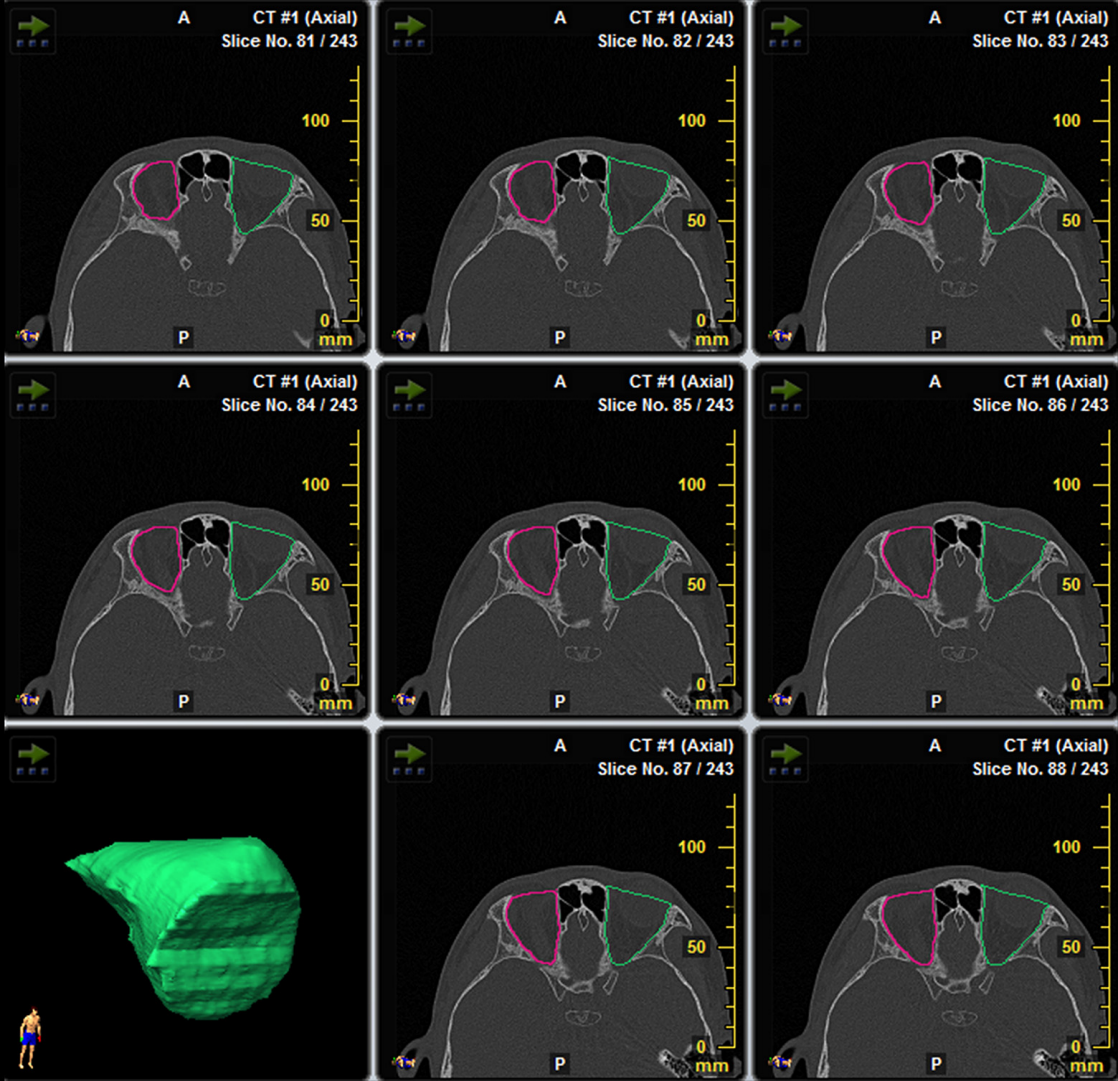
Measurement of orbital volume by iPlan Cranial software. The bony orbit was outlined in each slice of axial CT scans. The software generated 3D reconstruction of the orbit and calculated the orbital volume.

The bony orbit was outlined in each slice of axial CT scans. The anterior border of the orbit was defined as the line drawn between the inner aspects of the orbital rim on each side. At the level of medial canthi, the line was drawn between the lateral rim and the anterior lacrimal crest. The posterior border was a line that enclosed the foramens and fissures of the orbit. 3D volume rendering was implemented and orbital volume was calculated (Fig 1).

#### Global volume measurement

The globe was outlined in each slice of axial CT scans and the global volume was calculated by the iPlan Cranial software (same as the method used to measure the orbital volume)

#### Cyst volume measurement

The cyst was outlined in each slice of axial CT sans and the volume was obtained by the iPlan Cranial software (same as the method used to measure the orbital volume).

#### Pathlines

**Fig 2:**
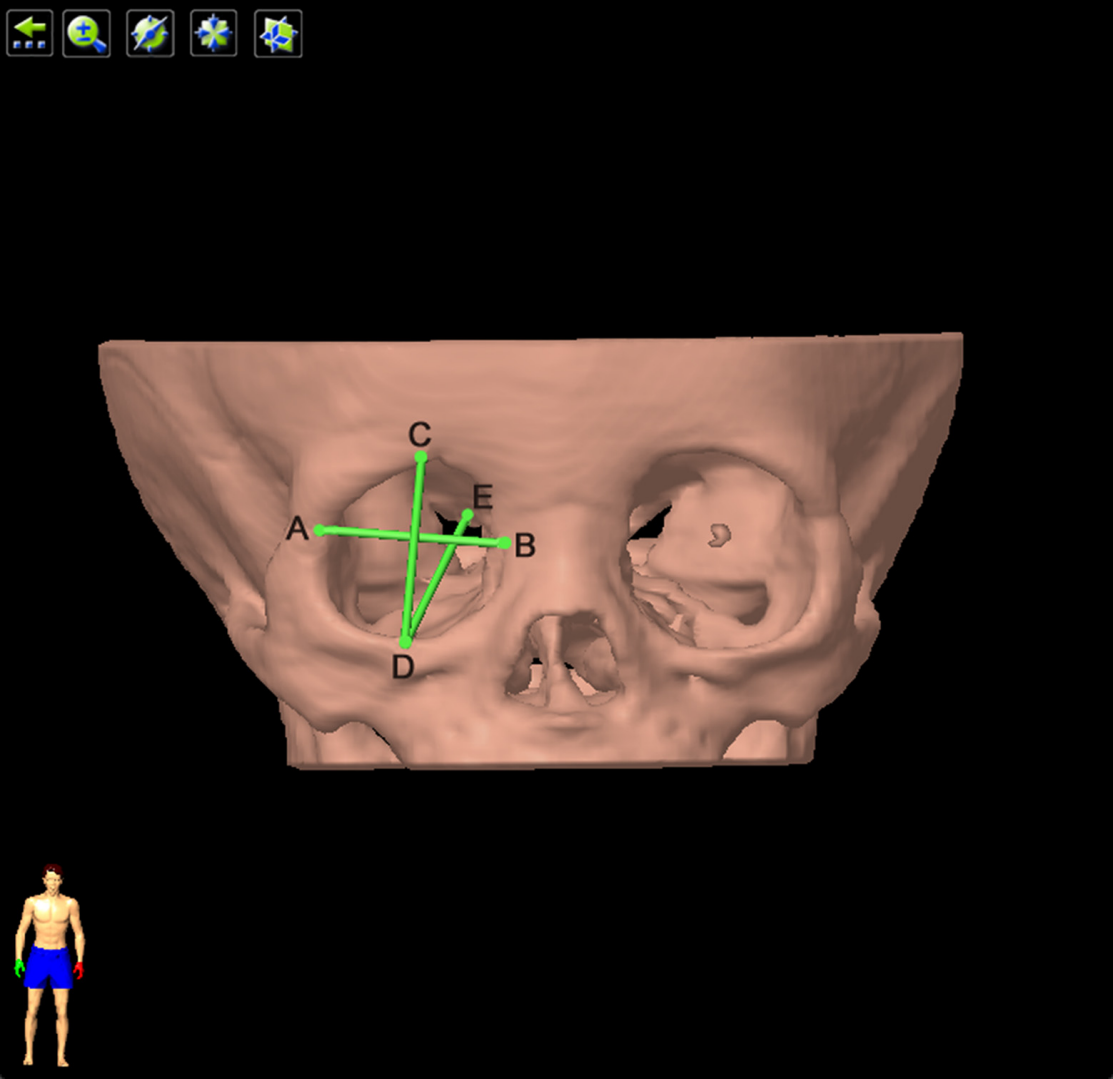
Measurement of orbital height, width and depth. Orbital width was the length of line AB. ‘A’ represented ectoconchion, and ‘B’ represented dacryon. Orbital height was the length of line CD. ‘C’, ‘D’ were the junctions of the midnormal of line AB with the superior and inferior orbital rims. Orbital depth was the distance between the lateral rim of the optic canal and the midpoint of the inferior rim. ‘E’ represented the lateral rim of the optic canal.

Orbital width was defined as the distance between dacryon and ectoconchion. The dacryon is the junction of the frontal bone, the lacrimal bone and the frontal tuber of the maxillary bone. The ectoconchion is the junction of the frontozygomatic suture and the curved surface of the orbital aditus. Orbital height was defined as the distance between the midpoints of superior and inferior orbital rims. The two midpoints were the junctions of the midnormal of the orbital width with the superior and inferior rims of the orbit. Orbital depth was defined as the distance between the lateral rim of the optic canal and the midpoint of the inferior rim (Fig 2).

**Fig 3:**
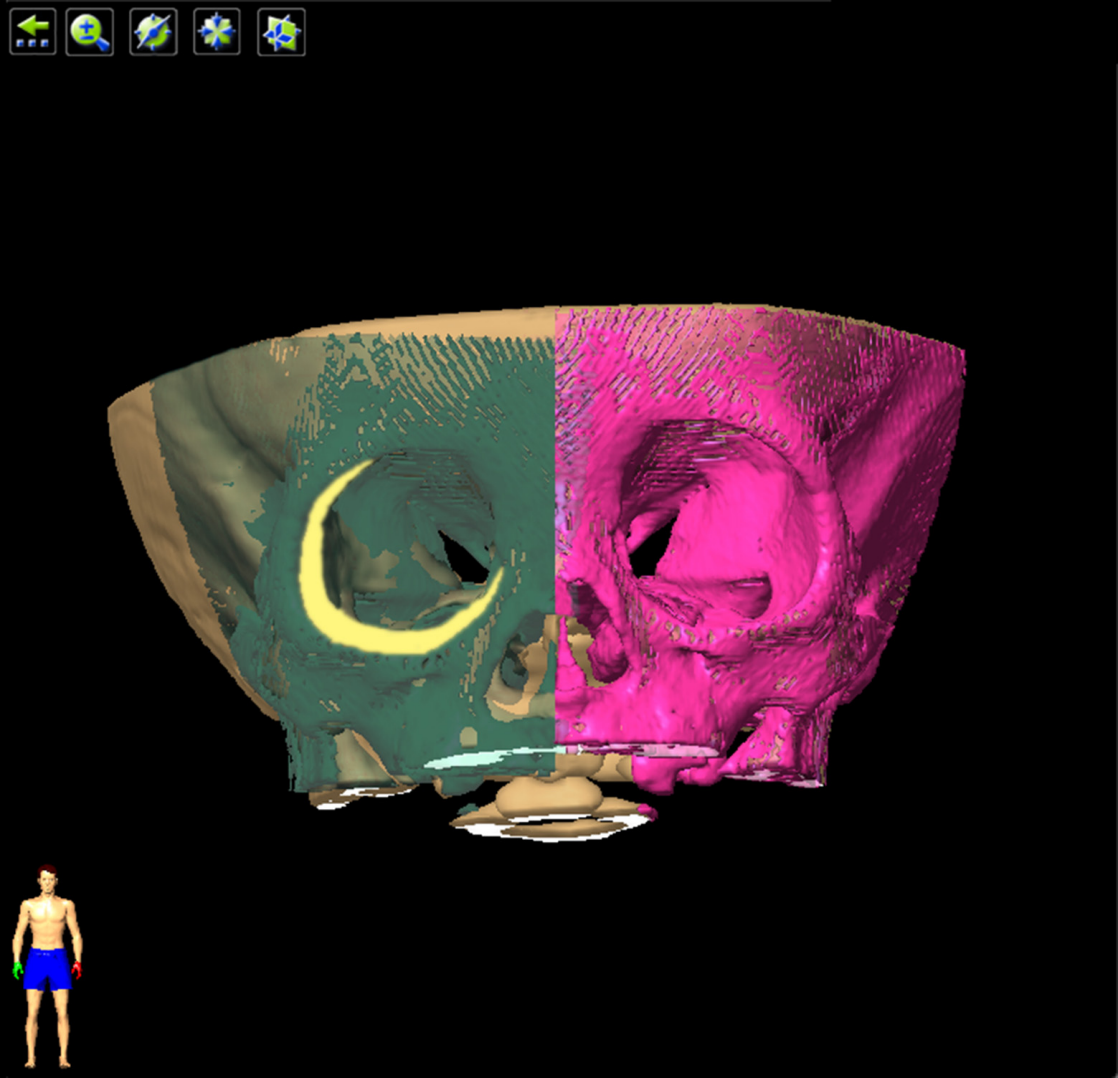
Measurement of orbital rim displacement. The axis of mirroring was the median sagittal plane. The purple area was the unaffected orbit, and the green area was the mirroring of the unaffected orbit. The yellow area demonstrates the difference between the affected and unaffected orbit. Displacement distance of superior midpoint, inferior midpoint, dacryon and ectoconchion represents the displacement of the superior, inferior, medial and lateral rims, respectively.

Displacement of the orbit rims was measured by comparing the mirror image of the unaffected orbit to the affected orbit. Displacement distance of superior midpoint, inferior midpoint, dacryon and ectoconchion represents the displacement of the superior, inferior, medial and lateral rims, respectively (Fig 3).

To minimize the influence of age, microphthalmic to contralateral ratio (MCR) of the orbital variables was studied instead of absolute values. MCR was defined as the ratio of the orbital variables of microphthalmic eye to the contralateral unaffected eye, including MCR of orbital volume, MCR of orbital width, MCR of orbital height and MCR of orbital depth.

### Analysis of the position of the cyst

#### Standard position

**Fig 4:**
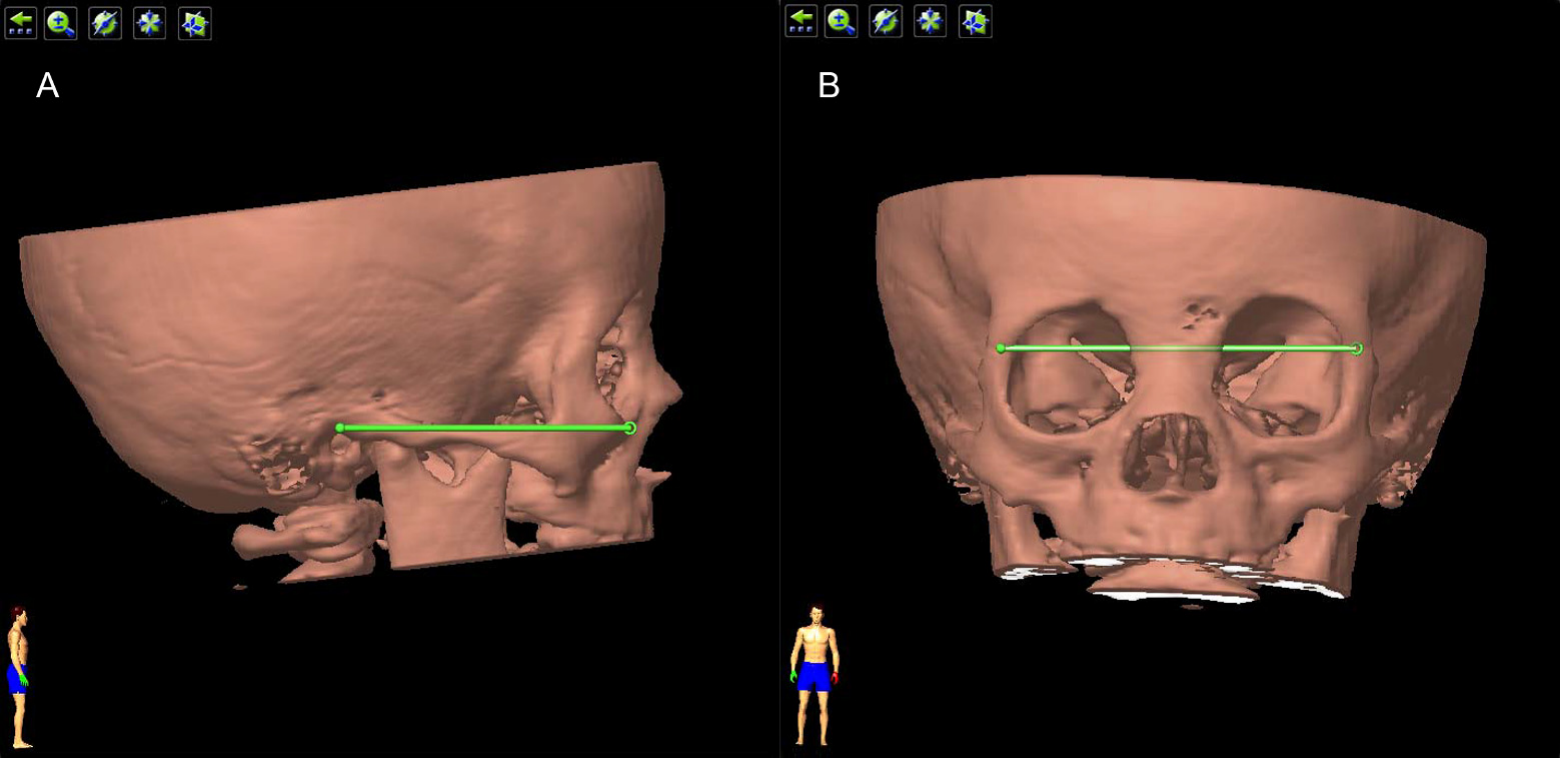
Standard position of the orbit. (A) The ‘standard lateral position’. The 3D image of the orbit was adjusted until both mandibles coincide and the Reid’s base line was horizontal. The Reid’s base line was the green line drawn from the inferior margin of the orbit to the superior margin of the orifice of the external acoustic meatus. (B) The ‘standard frontal position’. The orbit was rotated by 90° from standard lateral potion, and then adjusted until the line drawn between two frontozygomatic sutures (the green line) was horizontal.

To reach an unambiguous definition of the orientation of the reconstructed orbit, we used the following methods. First, on lateral position, the 3D image of the orbit was adjusted until both mandibles coincide and the Reid’s base line was horizontal. This was defined as “standard lateral position”. The Reid’s base line was the line drawn from the inferior margin of the orbit (Orbitale point) to the superior margin of the orifice of the external acoustic meatus (Auriculare point) (Fig 4A). After that, the orbit was rotated by 90°to frontal position, where we adjusted its position until the line drawn between two frontozygomatic sutures was horizontal. This was defined as “standard frontal position” (Fig 4B).

#### Definition of the centers

**Fig 5:**
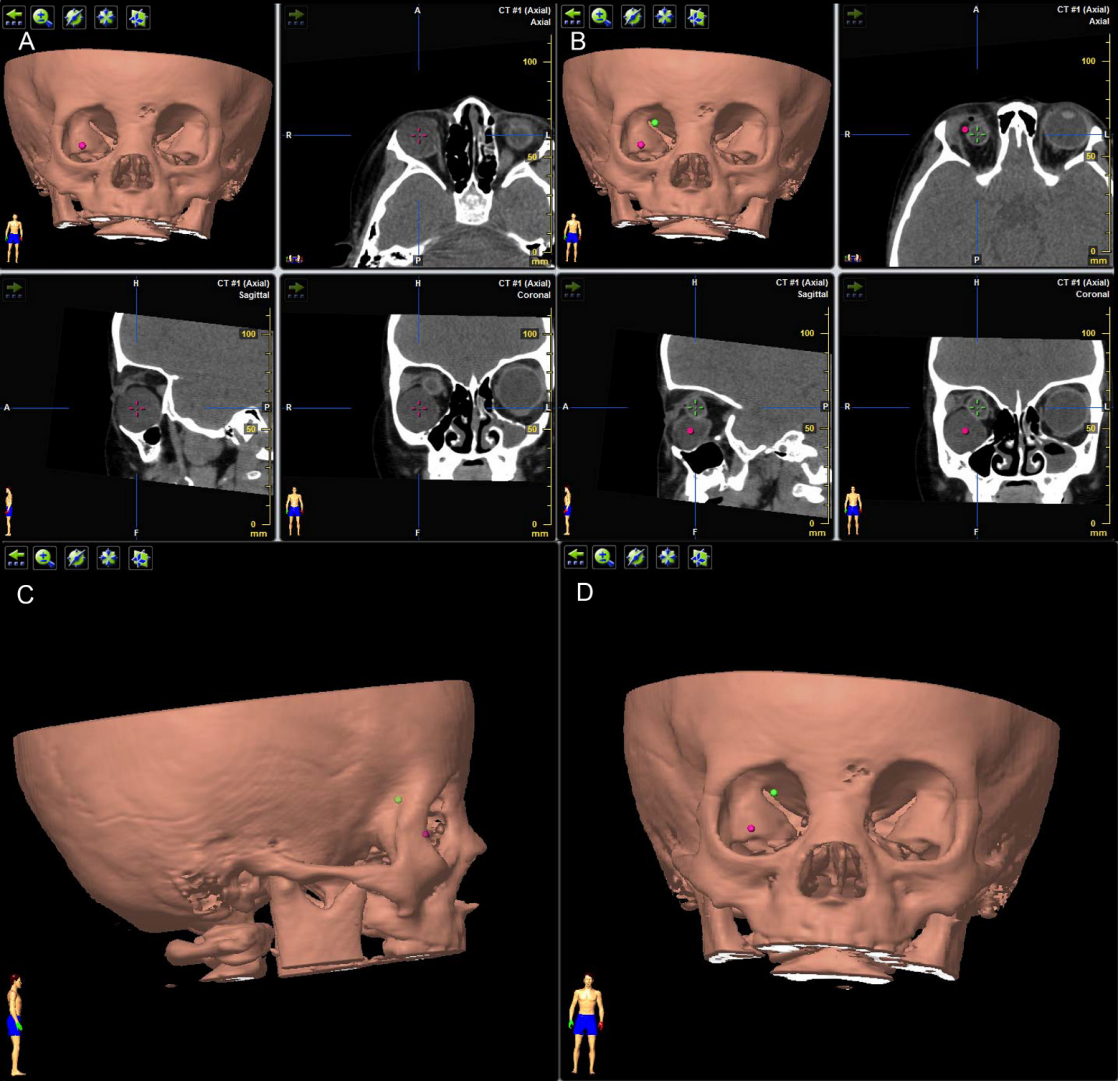
Identification of the position of cyst. (A) Center of the cyst was defined as the point located centrally on axial, sagittal and coronal views at the same time (purple cross). (B) Center of the globe was defined in the same way (green cross). Markings of the two centers were demonstrated simultaneously on 3D image of the orbit as purple and green dots. (C) On standard lateral position, the cyst (purple dot) was anterior to the globe (green dot). (D) On standard frontal position, the cyst located infratemporally to the globe.

To measure the relative position of the cyst, we need to define the centers of the cyst and globe. The center of the cyst was defined as the point located centrally on axial, sagittal and coronal views at the same time (Fig 5A). Center of the globe was defined in the same way (Fig 5B). Markings of the two centers were demonstrated simultaneously on the 3D image of the orbit (Fig 5B).

#### Position of the cyst

The position of the cyst was identified in three ways. First, on standard lateral position of the 3D image, by comparing the positions of the centers of cyst and globe, the cyst was defined as anterior to, posterior to or at the equator of the globe (Fig 5C). Second, on standard frontal position of the 3D image, the cyst was located as supranasal, infranasal, infratemporal, supratemporal or central to the globe (Fig 5D). Third, the cyst was identified as inside or outside the muscle cone by analyzing the CT images on different views by a same radiologist.

### Data analysis

Orbital parameters were compared between microphthalmic eyes with and without orbital cysts. Statistical analysis was conducted with SPSS 19.0 for Windows (SPSS Inc., Chicago, Illinois, USA). The normality test of numerical variables was conducted using the Kolmogorov-Smirnov test. Numerical variables that met the normal distribution were described as mean ± standard deviation and Student’s t test was used to compare differences between groups. Numerical variables that did not meet the normal distribution were expressed as median (25th percentile, 75th percentile) and the differences between groups were tested by Mann-Whitney U test. Qualitative data were described by frequencies and proportions and tested by Chi-square test or Fisher’s exact test. Correlation analysis was expressed as Pearson Correlation Coefficients. Two-sided p<0.05 was considered as statistically significant in all analyses.

## Results

### Demographics

Among the 20 patients with orbital cyst, 13 were males and 7 were females, age range was 0.67 to 32 years. The control group was composed of 53 males and 47 females; age range was 0.08 to 58 years. There was no statistical difference in gender (p = 0.325) or affected orbit (p = 0.742) between patients with cyst and the control group. However, significant difference was observed in ages of the two groups (p = 0.031) (Table 1). All of the patients with orbital cyst were unilateral. No systemic abnormalities were found in the patients.

**Table 1:** Demographic data of patients with cyst and controls.

		Controls (n = 100)	Patients with cyst (n = 20)	p value
Gender				0.325
	Male	53	13	
	Female	47	7	
Age, yr				
	Median(p25, p75)	4 (1, 13.75)	10(3.25, 27.75)	0.031^a^*
	Range	0~58	1~32	
Affected orbit				0.742
	Right	56	12	
	Left	44	8	

^a^ nonparametric test (Mann-Whitney U test).

*p<0.05.

### Volume and position of the cyst

The volume of the cysts ranged from 0.042ml to 7.551ml (1.234 (0.410, 3.598) ml). One patient (patient No. 16) had two cysts (both anterior to the equator; located infratemporally and supratemporally, respectively; cyst volume recorded as sum of the two cysts). The other 19 patients had only one cyst. 38.1% (8/21) of the cysts were anterior to or at the equator (anterior group). 61.9% (13/21) were posterior to the equator (posterior group). Cyst volume of anterior group was significantly larger than that of posterior group (p = 0.005). All of the cysts of anterior group were outside the muscle cone, while the majority (84.6% (11/13)) of the cysts posterior to the equator were inside the muscle cone. Among the 8 anterior cysts, 6 located infratemporally, 1 located supratemporally and 1 located infranasally. In the posterior group, 5 were right behind the optic nerve (central) and the rest 8 located in four different quadrants (Table 2).

**Table 2:** Volumes and positions of the orbital cysts.

		Anterior (n = 8)	Posterior (n = 13)	p value
Volume, ml (Median (p25, p75))		3.851(2.085, 6.575)	0.728(0.231, 1.766)	0.005^a^**
Location n(%)				0.064^**b**^
	Supranasal	0 (0.0)	2 (15.4)	
	Infranasal	1 (12.5)	2 (15.4)	
	Supratemporal	1 (12.5)	1 (7.7)	
	Infratemporal	6 (75.0)	3 (23.1)	
	Central	0 (0.0)	5 (38.5)	
Muscle cone n(%)				<0.001^b^**
	Inside	0 (0.0)	11 (84.6)	
	Outside	8 (100.0)	2 (15.4)	

^a^ nonparametric test (Mann-Whitney U test).

^b^ Fisher’s exact test.

**p<0.01.

### Comparison of MCR of orbital volume, MCR of orbital width, MCR orbital of height and MCR of orbital depth between microphthalmic patients with and without orbital cyst

There were significant differences between microphthalmic patients with and without orbital cyst in MCR of orbital volume (p<0.001), MCR of orbital height (p = 0.004) and MCR of orbital width (p = 0.043). For microphthalmic patients without cysts, the average volume of the affected orbit was 79.6% of the contralateral unaffected orbit, while for the patients with orbital cysts this ratio increased to 90.1%. The difference in MCR of orbital depth was not significant (p = 0.299) (Table 3). MCR of orbital volume had weak correlation with age (r = 0.221, p = 0.015). MCR of orbital height, MCR of orbital width or MCR of orbital depth were not correlated with age (r = 0.086, p = 0.352; r = 0.031, p = 0.738; r = -0.027, p = 0.768, respectively).

**Table 3:** Comparisons of MCRs and displacement of orbital rims between patients with cyst and controls.

		Controls (n = 100)	Patients with cyst (n = 20)	Increase/decrease	p value
MCR, % (mean±SD)					
	Volume	79.6±7.3	90.1±9.1	10.5	<0.001**
	Height	88.9±5.4	92.8±5.2	3.9	0.004**
	Width	90.7±5.4	93.3±4.8	2.6	0.043*
	Depth	95.6±3.4	96.5±3.7	0.9	0.299
Displacement, mm (Median (p25, p75))					
	Superior rim	1.50(1.10, 2.20)	1.35(0.55, 1.70)	0.15	0.042*
	Inferior rim	2.80(2.10, 3.50)	2.00(0.95,2.73)	0.80	0.001**
	Medial rim	0.30(0.30, 0.50)	0.30(0.23, 0.30)	0.00	0.021*
	Lateral rim	2.80(2.20, 3.70)	1.90(1.70,3.60)	0.90	0.049*

MCR, microphthalmic to contralateral ratio.

*p<0.05.

**p<0.01.

### Comparison of displacement of orbital rims between microphthalmic patients with and without orbital cyst

The orbital rims of microphthalmic eye were displaced compared with contralateral unaffected eye in microphtahlmic patients[13]. This displacement of orbital rims was decreased when an orbital cyst existed. The decreases of displacement of the superior and medial rims were 0.15mm (p = 0.042) and 0.00mm (p = 0.021), respectively, whereas those of the inferior and lateral rims were 0.80mm (p = 0.001) and 0.90mm (p = 0.049), respectively (Table 3). Displacement of superior, inferior, medial and lateral orbital rims had no correlation with age (r = -0.022, p = 0.814; r = -0.045, p = 0.629; r = -0.142, p = 0.122; r = -0.002, p = 0.985, respectively). We also tested the relationship between the displacement of the four orbital rims with age for infants (0~1y) and young children (1~6y) separately. Displacement of superior, inferior, medial and lateral orbital rims had no correlation with age for infants (r = 0.192, p = 0.300; r = -0.146,p = 0.434; r = 0.183, p = 0.325; r = 0.214, p = 0.247, respectively) or young children (r = 0.075, p = 0.650; r = -0.204, p = 0.213; r = -0.112, p = 0.498; r = -0.007, p = 0.965, respectively).

To avoid the influence of age, age-matched controls were selected randomly from each age group at a cyst/control ratio of 1:2.

### Demographic data of patients with orbital cyst and age-matched controls

The age-matched control group was composed of 17 males and 23 females; age range was 0.58 to 58 years, the meridian of age was 9.5(3.25, 21.75) years. There was no statistical difference in gender (p = 0.100), age (p = 0.742) or affected orbit (p = 0.713) between patients with cyst and the age-matched control group.

### Comparisons of MCRs and displacement of orbital rims between patients with cyst and age-matched control group

Significant differences were found between patients with orbital cyst and age-matched controls in MCR of orbital volume (p<0.001), MCR of orbital height (p = 0.032) and displacement of inferior orbital rim (p = 0.007). The differences in MCR of orbital width and MCR of orbital depth were not significant (p = 0.276 and p = 0.464, respectively) (Table 4). The decreases of displacement of the superior and medial rims were 0.15mm (p = 0.124) and 0.00mm (p = 0.147), respectively, whereas those of the inferior and lateral rims were 0.60mm (p = 0.007) and 0.60mm (p = 0.354), respectively (Table 4).

**Table 4:** Comparisons of MCRs and displacement of orbital rims between patients with cyst and age-matched controls.

		Age-matched controls (n = 40)	Patients with cyst (n = 20)	Increase/decrease	p value
MCR, % (mean±SD)					
	Volume	80.6±7.4	90.1±9.1	9.5	<0.001**
	Height	89.7±5.1	92.8±5.2	3.1	0.032*
	Width	91.7±5.8	93.3±4.8	1.6	0.276
	Depth	95.8±3.1	96.5±3.7	0.7	0.464
Displacement, mm (median (p25, p75))					
	Superior rim	1.50(1.10, 1.98)	1.35(0.55, 1.70)	0.15	0.124
	Inferior rim	2.60(2.13, 3.38)	2.00(0.95,2.73)	0.60	0.007**
	Medial rim	0.30(0.30, 0.50)	0.30(0.23, 0.30)	0.00	0.147
	Lateral rim	2.50(1.83, 3.40)	1.90(1.70,3.60)	0.60	0.354

MCR, microphthalmic to contralateral ratio.

*p<0.05.

**p<0.01.

### Correlation between cyst volume, globe volume and cyst-plus-globe volume for patients with orbital cyst

**Fig 6:**
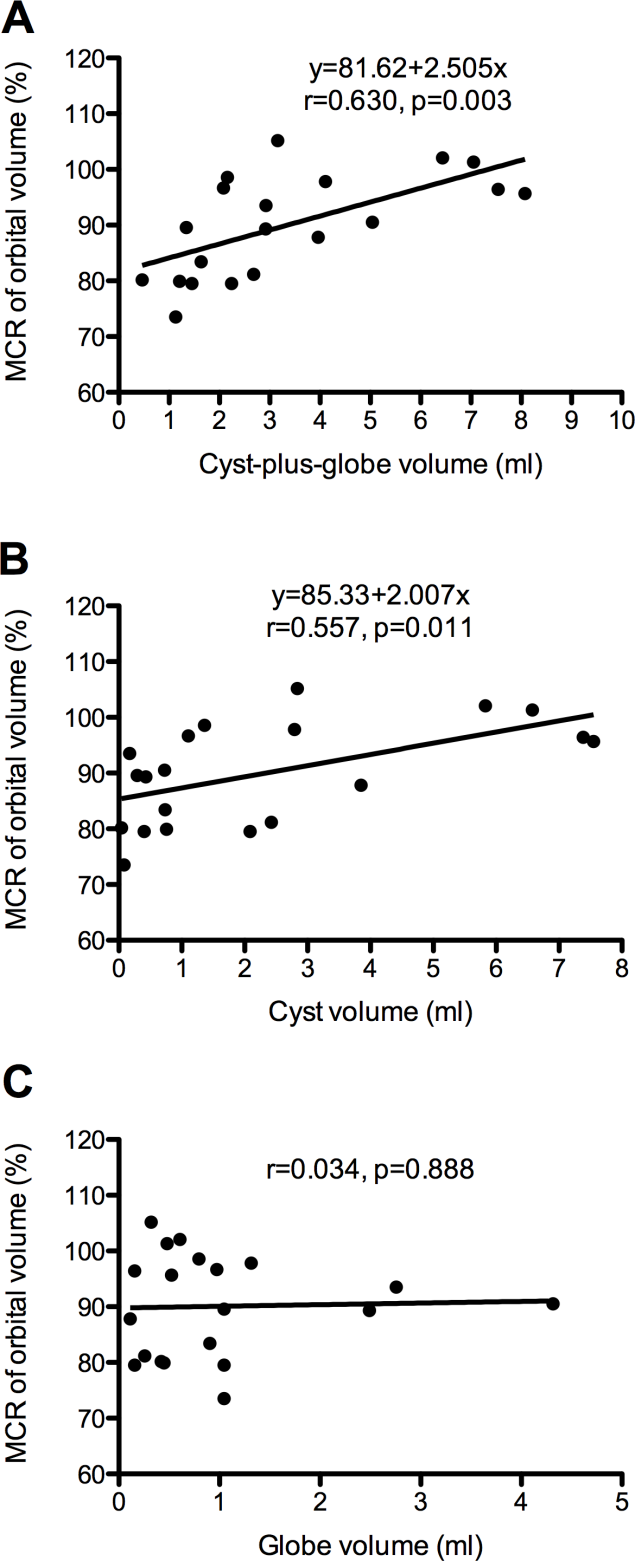
**Scatterplots showing the correlations between cyst-plus-globe volume (A), cyst volume (B), globe volume (C) (x-axis) and MCR of orbital volume (y-axis)**. The linear regression line (black line) was modified from the two variables.

For patients with orbital cyst, correlation analysis revealed that higher cyst-plus-globe volume was associated with higher MCR of orbital volume (r = 0.630, p = 0.003), cyst volume was also associated with MCR of orbital volume (r = 0.557, p = 0.011), but globe volume of the affected eye was not associated with any of the orbital parameters (Fig 6).

### Comparison of MCR of orbital volume, MCR of orbital width, MCR of orbital height, MCR of orbital depth and orbital rim displacement between patients with different cyst-plus-globe volume

We divided the patients with orbital cyst into two groups according to the cyst-plus-globe volume (cyst-plus-globe volume≥2.80ml as “large”, cyst-plus-globe volume <2.80ml as “small”). The median of cyst-plus-globe volume (2.80ml) was set as cut-off point when grouping patients in this study. There were significant differences between patients with large and small cyst-plus-globe volume in MCR of orbital volume (p = 0.002), MCR of orbital height (p = 0.014), MCR of orbital width (p = 0.005), MCR of orbital depth (p = 0.002) and orbital rim displacement in inferior (p = 0.004) orbital rim (Table 5). No statistical difference was found between patients with small cyst-plus-globe volume and the age-matched control group in MCR of orbital volume (p = 0.188), MCR of orbital height (p = 0.855), MCR of orbital width (p = 0.554), MCR of orbital depth (p = 0.156) or orbital rim displacement in superior (p = 0.534), inferior (p = 0.605), medial (p = 0.431) and lateral (p = 0.592) orbital rims.

**Table 5:** Comparisons between patients with small and large cyst-plus-globe volume.

		Large (≥2.80ml) (n = 10)	Small (<2.80ml) (n = 10)	Difference	p value
MCR, % (mean±SD)					
	Volume	96.0±5.8	84.2±8.1	11.8	0.002**
	Height	95.5±3.1	90.1±5.5	5.4	0.014**
	Width	96.1±3.0	90.5±4.6	5.6	0.005**
	Depth	98.8±2.7	94.2±3.1	4.6	0.002**
Displacement, mm (Median(p25, p75))					
	Superior rim	0.65(0.45, 1.53)	1.55(1.20, 1.73)	0.90	0.063
	Inferior rim	1.20(0.45, 2.00)	2.65(2.08, 3.28)	1.45	0.004**
	Medial rim	0.30(0.18, 0.35)	0.30(0.30, 0.33)	0.00	0.281
	Lateral rim	1.85(1.48, 2.05)	3.60(1.65, 4.15)	1.75	0.063

MCR, microphthalmic to contralateral ratio.

*p<0.05.

**p<0.01.

## Discussion

The prevalence rate of microphthalmos is about 1.5~1.8 per 10,000 births,[15, 16] and the prevalence rate of microphthalmos together with anophthalmos is about 1.0~3.21.[17–20] However, there is no agreed prevalence rate of microphthalmos with orbital cyst. Basically, it is very rare. In a survey composing 44 congenital microphthalmos, only 2 had associated orbital cysts.[21] In another large study of 29 patients with anophthalmos and 48 with microphthalmos, 3 patients had colobomatous cysts.[22]

Previous studies showed no obvious gender predilection of microphthalmos with orbital cyst.[11, 12] In our study, 65% of the patients were male. The condition can be unilateral or bilateral, but usually it is unilateral. In McLean et al.’s study involving 34 patients, 82.4% had unilateral lesion.[11] In Chaudhry et al.’s survey of 23 cases, 74% were unilateral.[12] However, there was no bilateral case in our study and 60% involved the right eye.

Microphthalmos with orbital cyst could be associated with abnormalities of different organs or systems, including cardiovascular system, central nervous system, urologic system, pulmonary, digestive tract and cleft lip/ palate.[11, 12, 23] Chaudhry et al. stated that the systemic abnormalities were more often associated with bilateral lesions than unilateral lesions.[12] McLean et al. found systemic abnormalities no more common in cases with bilateral cysts compared to cases with unilateral cysts.[11] Unlike other studies, in spite of consultation by a pediatrician, no systemic abnormalities were found in our study. The reason may be that patients with systemic disorders focused on treatment of life threatening diseases instead of seeing an ophthalmologist. Consultation by a pediatrician is mandatory to find any extra-ocular abnormalities in patients with newly diagnosed microphthalmos with orbital cyst.

In literatures, microphthalmos is assessed when the axial length, adjusted for age, is below the 95th centile in children or less than 18.5mm in adults.[24] This definition did not mention patients’ vision or the need of reconstruction. As oculoplastic surgeons, we paid more attention to improve patients’ looks instead of the exact axial length. In this study, we only included clinical blind microphthalmos, the definition of which was not quantifiable and had not been established by others. We defined it and all the microphthalmos in this study meant clinical blind microphthalmos.

Currently, CT scanning and magnetic resonance imaging (MRI) are the most popular methods to measure orbital volume. Compared to MRI, CT scanning has several advantages. 1) It is more economical and time saving. For children, it takes only 2~3 min to do a CT scan of the orbit, while an MRI examination needs 20~30min, which is too long for small children to coordinate even under sedation. 2) CT is outstanding in evaluation of skeletal structures including the orbit. It provides excellent image quality for bony landmarks. 3) CT provides abundant data for reconstruction of 3D image of the orbit and offers accurate and reproducible measurements. However, less exposure of radiation and higher resolution of soft tissue are superiorities of MRI. In our study, great attention was paid to the protection of children from radiation. The pediatric CT parameters were carefully optimized according to the ALARA principle[14] by reducing the peak kilovoltage and tube current and limiting the scan to the orbital region. Since highest-quality images were not necessary for measurement of orbital volume, some resolution could be compromised to minimize ionizing radiation risk. The characteristics and advantages of the iPlan Cranial software has been fully discussed in our previous paper.[13] It is rapid, accurate and reproducible when measuring the orbit. It also has the special function of mirroring, making it possible to measure the displacement of orbital rims.

The position and growth pattern of the cysts could be divided into two categories: 1) anterior and out of the muscle cone, 2) posterior and inside the muscle cone. 61.9% of the cysts located posterior to the equator. The anterior cysts tended to be larger than the posterior ones. Most of the anteriorly growing cysts located infratemporarily (6/8). Large cysts growing anteriorly usually appeared as bluish orbital masses expanding the inferior eyelid, causing severe cosmetic problems and making it impossible to wear prosthesis, as described in many case reports.[1, 2, 5, 6, 8, 9] The cysts locating retrobulbarly had much less influence on the conjunctiva sac and the eyelids.[3, 7] According to our research, the cysts have a predilection for retrobulbar space, but most of the reported cases were large anteriorly growing cysts. The reason may be that the anterior cysts are obvious clinically, while some posterior cysts could be neglected without high-quality imaging examination.

Because orbital volume and other orbital variables increase with age until the end of puberty, MCRs instead of the absolute values were compared between different groups to minimize the influence of age. Moreover, the final goal of treatment was symmetry of the two orbits, not the absolute volume of one side. Among the 120 participants, statistical analysis showed very weak correlation between age and MCR of orbital volume (r = 0.221, p = 0.015) and no correlation between age and all the other parameters. To further avoid the influence of age, we selected an age-matched control group and got similar study results.

The orbit is supposed to develop with the craniofacial skeleton from birth to teenage hood. However, the small, hypoplastic globe in microphthalmic children is inadequate to stimulate the normal growth of orbit and results in hemifacial deformity, causing severe cosmetic problems and psychological challenges in the patients. Pressure was found to be an effective stimulant in expansion of craniofacial skeleton. Orbital implants designed to stimulate orbital bone growth and socket enlargement have been proved to be effective.[25–30] Tse et al. tested the efficacy and biocompatibility of a serially inflated orbital tissue expander to stimulate bone growth in an anophthalmic feline model.[28] Their further study in congenital anophthalmic children also showed that the orbital tissue expander was safe and effective in stimulating anophthalmic socket bone growth. [29] Microphthalmos with orbital cyst is a special variant of microphthalmos, with the cyst serving as natural volume replacement. Orbital cyst might play a positive role in orbital volume expansion. According to our research, the MCR of orbital volume increased from 79.6% in controls to 90.1% in patients with orbital cyst. MCRs of orbital height and orbital width also increased significantly. The asymmetry between the affected and unaffected orbits was smaller in patients with orbital cyst than those without it.

Our previous study demonstrated that for microphthalmic children, the decrease in orbital volume resulted from decrease in the cross-sectional area of the orbital aditus (determined by orbital height and width), rather than the orbital depth.[13] In the present study, the difference of MCR of orbital depth between patients with cyst and controls was not significant either, but the differences of MCRs of orbital height and width were significant. This indicates that orbital cyst might stimulate the expansion of the orbital aditus rather than orbital depth.

Studies have demonstrated that orbital enlargement effect is proportional to the volume implanted. Considering that the orbital contents were composed of the cyst as well as the microphthalmic globe, we took into account the globe volume. According to our research, the cyst-plus-globe volume was positively correlated with MCR of orbital volume. Moreover, there were significant differences in MCRs of orbital volume, height, width and depth between patients with large and small cyst-plus-globe volume, but the differences were not significant between patients with small cyst-plus-globe volume and controls, which indicate only large cyst was associated with better developed orbit.

Patients with orbital cyst also showed decreases in displacement of orbital rims, especially the inferior and lateral rims (by 0.80mm, p = 0.001 and 0.90mm, p = 0.049, respectively). This may be due to the fact that most of the cysts were attached to the inferior portion of the globe (the location of the fetal fissure). According to our previous research, the retardation of orbital development in microphthalmic patients was mainly revealed in that of inferior and lateral rims.[13] When an orbital cyst existed, the retardation was also alleviated most remarkably in these two rims. Significantly less displacement of inferior orbital rim was also observed in patients with larger cyst-plus-globe volume. The infratemporal growth pattern of most of the large cysts may explain this result to some extent.

Currently, there is no consensus on the treatment strategy for microphthalmos with orbital cyst. Duke Elder[31] described three categories of cyst associated with microphthalmos: 1) a relatively normal eye with a small cyst, which is not apparent clinically; 2) an obvious cyst associated with a grossly deformed eye; 3) a large cyst which has pushed the globe backwards so that it is not visible clinically. When the cyst is very small and not apparent clinically, we focus on socket expansion and do watchful observation on the cyst. When the cyst is obvious but dose not interfere with prosthesis wearing, we also keep the cyst, because any small or medium sized cyst (together with the globe remnant) could stimulate orbital expansion.[11] Actually, if the cyst does not disturb prosthesis wearing, there is not much difference in the treatment of micropthalmic patients with orbital cyst and patients without orbital cyst. Conformers are used as early as possible. In cases where orbital volume is poor other volume expansion techniques should be considered if conformer therapy is inadequate on its own. Whether a cyst prevents the use of prosthesis not only depends on its size but also on its position and growth pattern. We have seen very large cysts that were confined within the orbit and did not protrude, leaving enough space for the prosthesis. In such circumstances, there is no need to excise the cyst. We only have to excise the cyst when prosthesis cannot be fitted in the conjunctival sac or when the cyst prolapses from the palpebral fissure. According to our data, 61.9% of the cysts grew posteriorly, doing no harm to the configuration of conjunctival sac or eyelid. Actually, only two patients had cyst excisions because of their disturbance to prosthesis fitting. We replaced the orbital volume with hydrogel expanders and got satisfying cosmetic outcome.

There are some limitations of this study. Since micropthalmos with orbital cyst was a very rare condition, the sample size of this study was small. There were not enough patients in each age group for us to analyze the influence of orbital cyst on orbital development. From this cross-sectional study, we could reach the conclusion that patients with orbital cyst had better developed orbits, but we could not interpret the cause and effect between the cyst and the development of orbit. Long-term observations on the orbital development should be carried out in the future.

In conclusion, by comparing parameters including MCRs and displacement of orbital rims, we found that microphthalmic eyes with large cyst-plus-globe volume had better similarity with the contralateral eyes, comparing with microphthalmic eyes without orbital cyst or with small cyst-plus-globe volume. This might indicate the positive role of orbital cyst (especially large cyst) in orbital development of microphthalmic patients.

## Supporting Information

S1 File Data of Patients with Cyst. (XLS)

S2 File Data of Control Group. (XLSX)

S3 File Data of Age-matched Controls. (XLSX)
